# Magnetic properties in α-MnO_2_ doped with alkaline elements

**DOI:** 10.1038/srep09094

**Published:** 2015-03-13

**Authors:** Li-Ting Tseng, Yunhao Lu, Hai Ming Fan, Yiren Wang, Xi Luo, Tao Liu, Paul Munroe, Sean Li, Jiabao Yi

**Affiliations:** 1 School of Materials Science and Engineering, University of New South Wales, Kensington, 2052, NSW, Australia; 2 School of Material Science and Engineering, Zhejiang University, 310027, China; 3 Shanxi Key Laboratory of Degradable Biomedical Materials, School of Chemical Engineering, Northwest University, Xi'an, Shanxi 710069, China; 4 Institute for Synchrotron Radiation. Karlsruhe Institute of Technology, Karlsruhe, Germany

## Abstract

α-MnO_2_ nanotubes were fabricated using a hydrothermal technique. Li, Na and K ions were introduced into MnO_2_ nanotubes to tailor their magnetic properties. It was found that with a doping concentration lower than 12 at%, the nanotubes showed ferromagnetic-like ordering at low temperature (<50 K), while antiferromagnetic coupling dominated their physical behavior with doping concentrations beyond 12 at%. Such experimental phenomenon was in very good agreement with associated first principle calculations. The ferromagnetic-like ordering originates from the breaking of equivalence between two different Mn-O octahedrals in α-MnO_2_ due to the filling of alkaline ions in the tunnels. Both small charge transfer and lattice distortion play important roles in the ferromagnetic ordering.

Manganese dioxide, MnO_2_, has been widely used as catalysts, cathodes of lithium batteries and sieves in industry due to its unique physical and chemical properties, as well as its relative abundance in nature^1^,^2^,^3^,^4^,^5^,^6^. As the precursor material for the lithium battery, an important feature of MnO_2_ is the presence of mesoporous channels formed by the stacking of MnO_6_ octahedrons, which can host K, Na, or Li ions. The framework of these mesopore channels, called octahedral molecular sieve structures, can form infinite alternating 1 × 1 and 2 × 2 tunnels. β-MnO_2_ has 1 × 1 tunnels, whilst α-MnO_2_ has 2 × 2 tunnels by sharing the edges and corners of the MnO_6_ octahedrons^7^,^8^. Detailed studies have shown that many elements can be doped into the structures due to the relatively large voids of these tunnels, such as cations of alkaline, alkaline earth elements, as well as heavy metals^9^,^10^,^11^.

The properties of α-MnO_2_ can be tuned for practical applications through careful selection of doping elements. It is well known that the Li ion has a small atomic radius (0.076 nm), in comparison with the size of the tunnels (0.48 nm). As such, a cluster of Li ions can be housed in the tunnels and can migrate freely in these tunnels under electrochemical stimulus. Such physical behaviour is promising for many applications such as supercapacitors and batteries^2^,^12^,^13^,^14^,^15^,^16^,^17^.

The ground state of α-MnO_2_ is antiferromagnetic due to the symmetric nature of Mn-O-Mn bonds. However, α-MnO_2_ nanorods prepared through a hydrothermal method using KMnO_4_ as the precursor have been discovered to show ferromagnetic-like behaviour at very low temperatures (i.e. 5 K)^18^,^19^,^20^,^21^,^22^. Furthermore, the saturation magnetization or coercivity can be tuned by varying the doping concentrations of K ions. It has been experimentally shown that a lower concentration of K ions could induce stronger such ferromagnetism. When the doping concentration of K^+^ is greater than 15 at%, the ferromagnetic-like behaviour disappears due to the appearance of antiferromagnetism. The mechanism of this phenomenon still remains unclear. The geometrical frustration on the triangular lattices and the mixture of Mn^3+^ and Mn^4+^ ions have been considered to be attributed to the ferromagnetic-like properties^19^. However, there is no clear experimental evidence to support this hypothetical suggestion. Density function theory (DFT) calculations on K-doped MnO_2_ have shown that Mn^3+^ is formed due to the electron transfer for the K^+^ doping, which results in the overlap of the Fermi level with the conduction band, leading to metallic behaviour^23^. However, Mn^3+^ has never been observed and the magnetic properties of K-MnO_2_ by DFT calculation have never been reported. Since the tunnel is as large as 0.48 nm in diameter, the doping from K^+^ may not change the geometry significantly at low doping concentrations. Furthermore, antiferromagnetism appears in these samples when the doping concentration of K^+^ is higher than 15 at%. This suggests that the lattice distortion alone may not be the origin of ferromagnetic-like properties^18^. So far, there are many contradictions in the proposed mechanisms and observed phenomena. Therefore, a more detailed investigation is needed to identify unambiguously the mechanism generating such ferromagnetic-like behaviour in this material.

In this work, we studied the ferromagnetic behaviour in K-doped MnO_2_ nanotubes. X-ray absorption near edge spectroscopy (XANES) indicates that Mn is very close to 4+ valency rather than 3+, suggesting very weak charge transfer and this weak charge transfer alone may not be the origin of ferromagnetic-like behaviour. Detailed investigations reveal that neighbouring tunnels which may be filled and unfilled lead to differences in the magnetic moment of Mn atoms in the apex site and plane site, thus breaking the balance of Mn-O-Mn bonds. This results in the ferromagnetic-like ordering. Furthermore, we can also fill Li or Na ions into these tunnels by exchanging Li/Na ions with K^+^ and this leads to filling behaviour and induced ferromagnetic properties that are similar to that observed by K^+^ doping. α-MnO_2_ is one of the most promising anode materials for lithium batteries. However, the existence of K^+^ during fabrication has impeded charging/discharging of these batteries. This work has shown that Li or Na ions can totally replace K^+^ through a solution exchanging method. The understanding of Li or Na filling mechanism in MnO_2_ may be of importance for the development of high performance batteries.

## Results and Discussion

### Characterization of MnO_2_ nanorods

We first examine the X-ray diffraction (XRD) patterns of K-doped MnO_2_ nanotubes. This demonstrates that the phase of all the samples is α-MnO_2_ and no secondary phase is observed (Fig. S1a). In addition, the intensities of the peaks for 16 at% K-doped MnO_2_ is much lower than that of the others, suggesting disordering in this sample due to the high concentration of K doping. If we enlarge the (211) peak, which has the strongest intensity (Fig. S1b), we can see that with increasing doping concentration, the peaks shift to a lower 2θ value, suggesting d spacing expansion caused by the incorporation of K in the tunnel. The XRD spectra of the Li and Na-doped MnO_2_ are similar to that of K-doped MnO_2_. No secondary phases were observed in either sample.

**Figure 1:**
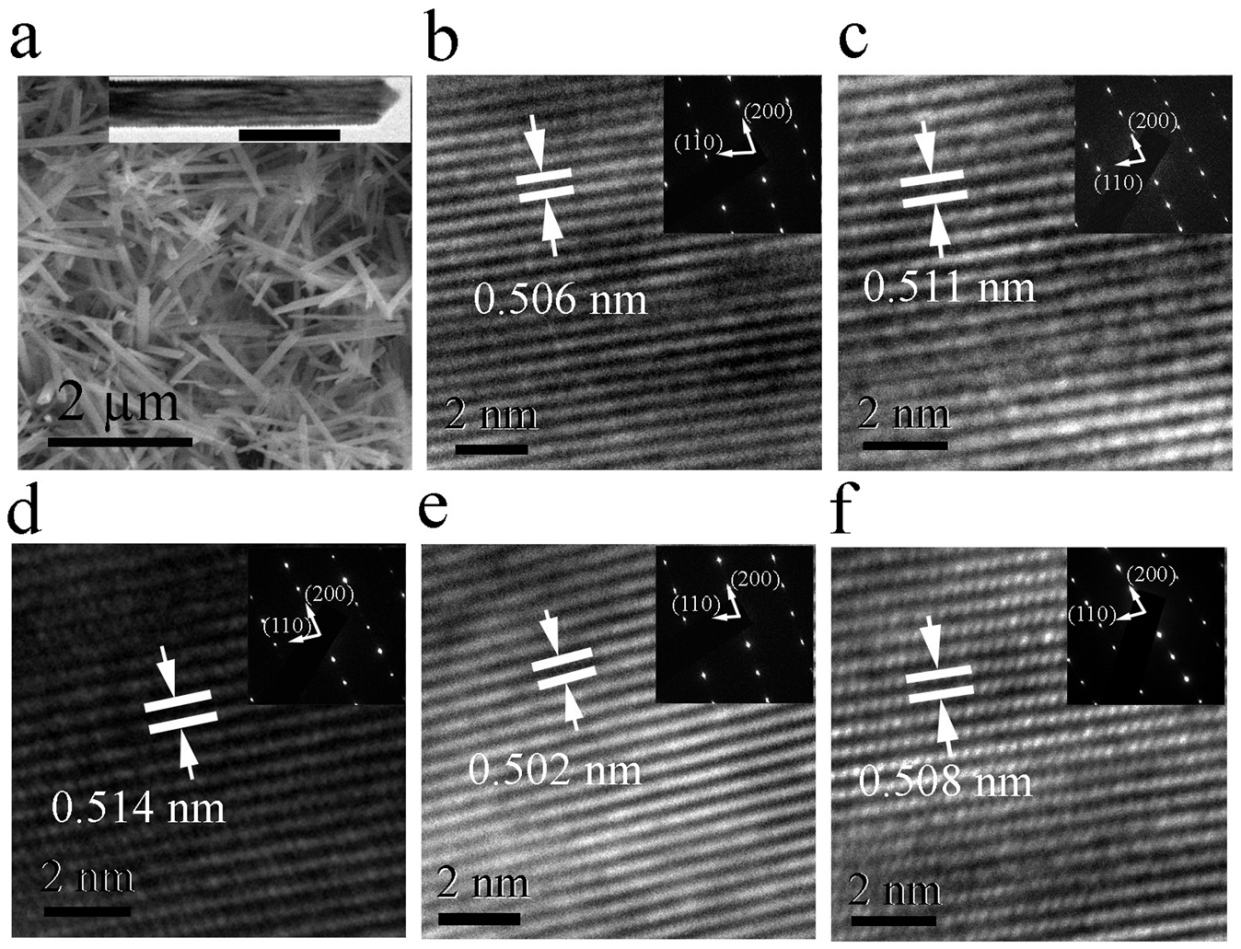
SEM and TEM images of alkaline element doped MnO_2_. (a) SEM image of α-MnO_2_ nanotubes. The inset is the TEM image of a single MnO_2_ nanotube at low magnification. The data bar is 100 nm. (b) – (f) are high resolution TEM images in the (200) plane of the 2 at%, 6 at%, 12 at% K-doped MnO_2_, 6 at% Li and 6 at% Na doped MnO_2_ respectively. The insets are SAED patterns of the corresponding samples.

To further verify the effects of doping, Transmission electron microscopy (TEM) analysis was used to investigate the microstructure of the doped oxides as shown in Fig. 1. The shape of the nanotubes is similar to that in the literature (Fig. 1a)^19^ The inset of Fig. 1a shows a typical square shaped MnO_2_ nanotube under low magnification TEM imaging. All the tubes with doping concentrations lower than 12 at% show strong crystalline structures as seen from Fig. 1b–1e. Whereas, the high resolution TEM image of 16 at% K-doped MnO_2_ exhibits some localised disordering (Fig. S2), which is consistent with the XRD measurements. D-spacing analysis using digital micrograph software indicates that 2 at%, 6 at% and 12 at% K-doped MnO_2_ samples have a d-spacing values of 0.506, 0.511 and 0.514 nm in the (200) plane of α-MnO_2_ respectively (Fig. 1b to 1d). This indicates that K doping leads to lattice expansion and the higher the doping concentration the greater the lattice expansion, confirming that introducing K ions into MnO_2_ results in geometry change. Li and Na-doped MnO_2_ have a lattice spacing of 0.510 and 0.512 nm in the (200) plane, respectively, which are smaller than that of K-MnO_2_ with 6 at% K doping. This may be due to the relatively lower dopant concentration and relatively smaller radii of Li^+^ (0.9 nm) and Na^+^ (0.116 nm) (Fig. 1e and 1f).

**Figure 2:**
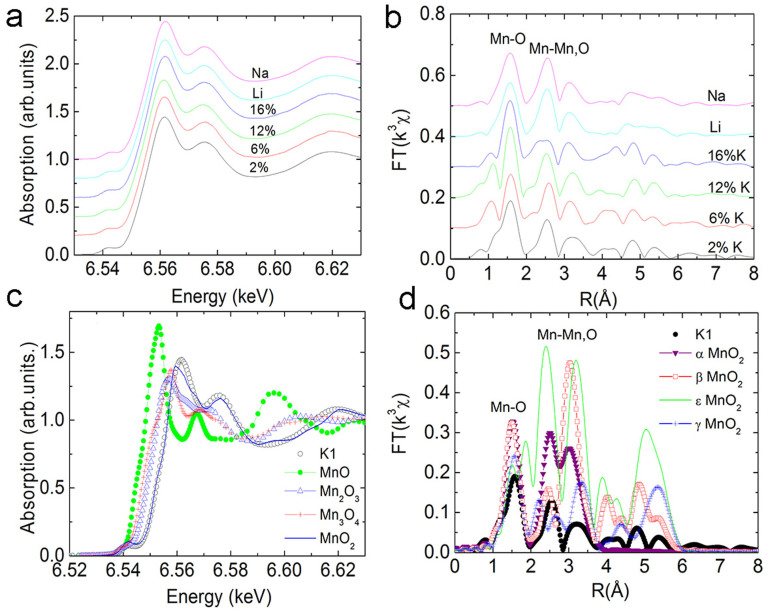
XAFS of alkaline element doped MnO_2_. (a) XAFS spectra of K-MnO_2_ at different doping concentrations and Li/Na doped MnO_2_; (b) Fourier transform of the XAFS data in (a); (c)XAFS in the near edge of different forms of manganese oxides; (d) Fourier transformation of 2 at% K-doped MnO_2_ and MnO_2_ at different phases.

As discussed, the mixture of Mn^3+^ and Mn^4+^ valence state in α-MnO_2_ has been considered as one of the reasons for the ferromagnetic ordering/spin glass behaviour at low temperature^18^. To determine the valence state of Mn in doped MnO_2_, X-ray absorption fine structure (XAFS) measurements were performed for all the samples, as shown in Fig. 2. From the XAFS spectra in Fig. 2a, it can be seen that all the spectra have similar chemical shifts, which corresponds to the presence of Mn^4+^. No trace of Mn^3+^ species can be observed in the spectra. Fig. 2b shows the XANES spectra of the 6 at% K-MnO_2_ compared to other forms of manganese oxide, such as MnO, MnO_2_, Mn_2_O_3_ and Mn_3_O_4_. It is found that the XANES of the 6 at% K-MnO_2_ in the near edge overlaps with that of MnO_2_, indicating a Mn^4+^ valence state for this sample. These results indicate that charge transfer between K and Mn is very weak, which is not readily detected by XAFS analysis.

Fourier transform of the XAFS data and the fitting to the first Mn-O shell indicate that 2% K-MnO_2_ has very small distortion in its structure (Fig. 2b). 6 at% and 12 at% K-MnO_2_ has an eminent distortion in the first Mn-O shell (Table SI in the supporting information), suggesting that the inclusion of K in the tunnels induces geometric frustration of the triangular lattices, which may destroy the balance of Mn-O-Mn antiferromagnetic ordering, leading to ferromagnetic-like ordering. Though Jahn-teller distortion due to the existence of Mn^3+^ has been proposed in MnO_2_^23^,^24^, in this work, XAFS cannot detect Mn^3+^ in these samples. From the structure of α-MnO_2_, the tunnel has a diameter larger than 0.48 nm. A small amount of K doping should not induce lattice distortion. It has been reported that the tunnels in α-MnO_2_ are usually supported by doped ions to avoid the collapse of these structures^23^. In our experiments, the K concentration has been strongly diluted by HCl exchange. Whilst a collapse of structure was not observed, suggesting that the OH^−^, H_2_O or H_3_O^+^ may always act to support the scaffold^9^,^25^. Therefore, a small amount of K doping may still induce some degree of lattice distortion. For samples with a doping concentration higher than 16 at%, there is a small distortion in MnO_6_ octahedrons of the first shell, but a large distortion in the high order shells, similar to that of a short range ordered material (Debye Waller factor 0.0114 from Table S1 in the supporting information). It is known that the α-MnO_2_ has 12.5 at% tunnels, which may be fully filled if the concentration of K ions is higher than 12.5 at%. This uniform distribution may reduce the extent of distortion in MnO_6_. Certainly, some tunnels may be filled with more than one K ion, leading to small distortion. While, some other K ions may reside in the interstitial sites of MnO_6_ rather than tunnels, producing a more disordered structure. Similarly, 6 at% Li or Na doping does not induce a large distortion in the first MnO_6_ shell. This is due to the relatively lower dopant concentration of Li and Na. The small atomic radius of Li^+^ (0.09 nm) and Na^+^ (0.116 nm) may also explain the smaller distortion in MnO_6_.

MnO_2_ is polymorphic with α, β, ε and γ phases. Fig. 2d shows the Fourier transform spectra of both 6 at% K-MnO_2_ and standard traces for the α, β, ε and γ phases. From the comparison of Mn-O and Mn-Mn shells, it confirms that all the nanotubes fabricated in this work are α-MnO_2_ and this is consistent with the XRD analysis.

### Magnetic properties measurement

**Figure 3:**
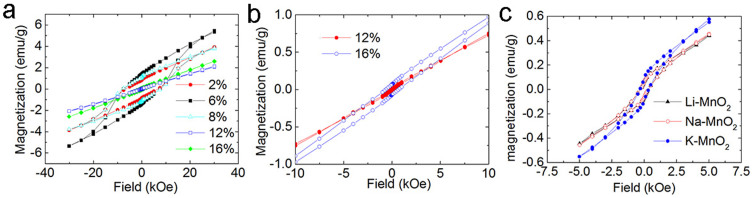
M-H loopes of Li, Na, K doped MnO_2_ nanotubes. (a) M-H loops of K-MnO_2_ with different doping concentrations; (b) Narrow scale of M-H loops of 12 at% and 16 at% K doped MnO_2_; (c) M-H loops of Li-MnO_2_, Na-MnO_2_ and K-MnO_2_ taken at 40 K.

Ferromagnetic-like behavior of K-MnO_2_ has been observed in previous studies^18^. In this work, we found that when K doping concentration is lower than 12 at%, the hysteresis loop with eminent coercivity appears at 5 K, indicating ferromagnetic ordering at low temperatures (Fig. 3a). 6 at% K-MnO_2_ presents the highest saturation magnetization (5.2 emu/g at 30 kOe) and coercivity (7500 Oe). The mechanism for the high coercivity is not very clear. It may be associated with the exchange coupling between antiferromagnetic phase and ferromagnetic-like phase as that in NiO nanostructures^26^,^27^. The decreasing coercivity with increasing doping concentration may be due to the increasing antiferromagnetic phase with small ratio of ferromagnetic-like phase^26^,^28^,^29^. It is interesting to note when the doping concentration is higher than 12 at% a linear M-H curve is observed and the magnetization is much smaller than that of MnO_2_ with the lower doping concentrations. This suggests that the sample may become antiferromagnetic again. In order to study the M-H curves in detail, we enlarged the M-H curves for 12 at% and 16 at% K-MnO_2_ over a narrow scale as shown in Fig. 3b. For the 12 at% K-doped MnO_2_, the curve is almost linear, suggesting the antiferromagnetic signal in dominant. However, a small coercivity (25 Oe) can still be detected in the sample, suggesting very weak ferromagnetic-like ordering. For the 16 at% K-doped MnO_2_, a coercivity of nearly 650 Oe was observed, indicating relatively strong ferromagnetic-like ordering in this sample in addition to the more dominant antiferromagnetic signal. This results suggest that ferromagnetic-like behavior may not only originate from the electron charge transfer by K doping and also the Mn^3+^ related to Jahn-teller distortion as the increase of K doping concentration should lead to more charge transfer, thus enhances the ferromagnetic-like ordering if the ferromagnetic-like behavior is arisen from charge transfer alone. However, it has been discovered that 12 at% K doped MnO_2_ has a very weak ferromagnetic-like ordering compared to that of 6 at% K doped MnO_2_. It should be noted that we did not measure pure α-MnO_2_ nanotubes without K, Li or Na doping since it is impossible to achieve totally alkaline element free MnO_2_ tubes with a chemical synthesis technique using KMnO_4_ as a precursor. Fig. 3c shows the hysteresis loops of 6 at% K-MnO_2_, 6 at% Li-MnO_2_ and 6 at% Na-MnO_2_ taken at 40 K, respectively. K-MnO_2_ has the highest saturation magnetization among the samples at the same doping concentration. While Li and Na doped MnO_2_ nanotubes have similar saturation magnetization and coercivities. The M-H curves of 6 at% Li-MnO_2_ and 6 at% Na-MnO_2_ measured at 5 K are shown in supporting information Fig. S5. The coercivities in these two samples are comparable to that of 6 at% K-MnO_2_.

Since Mn impurities, such as Mn_3_O_4_, is ferrimagnetic, which may contribute to the magnetic ordering in MnO_2_ nanorods. Our TEM, XRD and EXAFS have not detected any impurities phases other than α-MnO_2_ nanotubes. The resolution is better than 1%. From Ref. 30, the saturation magnetization of Mn_3_O_4_ is around 20 emu/g. Hence, the contribution from Mn_3_O_4_ is only 0.2 emu/g, which is negligible for the sample with a saturation magnetization of 5.2 emu/g. In addition, the Curie temperature of Mn_3_O_4_ nanoparticles is around 40 K, while in our work, the Curie temperature is around 50 K. Furthermore, by increasing or decreasing alkaline doping concentration, the magnetization and coercivity will vary accordingly, supporting that the magnetization in our samples are not from Mn_3_O_4_ impurities.

**Figure 4:**
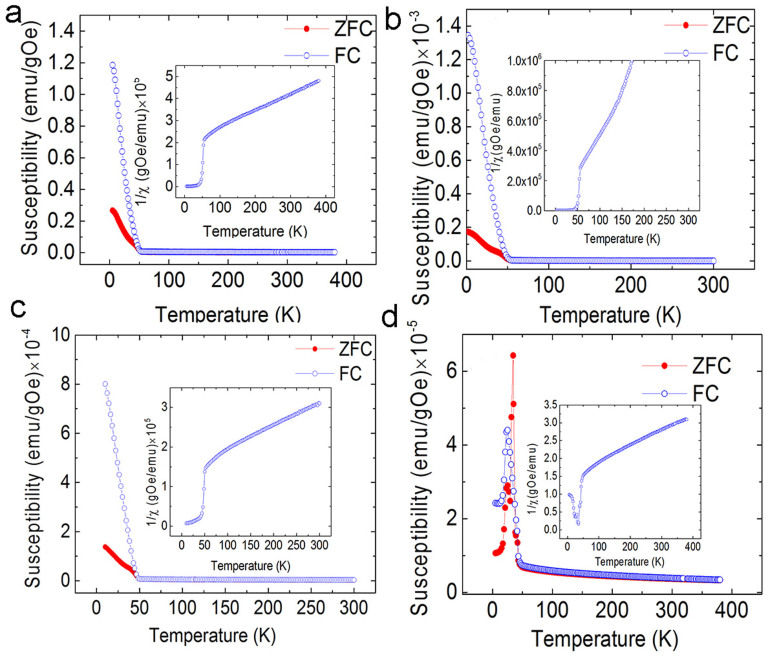
ZFC and FC curves with different doping concentrations. The applied field is 50 Oe. (a) 2 at%; (b) 6 at%; (c) 8 at%; and (d) 12 at% K-doped MnO_2_. The inset is the reverse susceptibility of corresponding samples.

From zero field cooling/field cooling (ZFC/FC) measurements, the critical temperature of ferromagnetic-like ordering was measured as shown in Fig. 4. 2 at% K-doped MnO_2_ has a Curie temperature of 50.4 K. The reverse susceptibility indicates that the nanotubes also have a negative susceptibility, which indicates the samples have a mixture of ferromagnetic-like and antiferromagnetic phase. It is known that pure MnO_2_ is antiferromagnetic. Hence, the antiferromagnetic signal should come from antiferromagnetic MnO_2_ itself. The ferromagnetic-like signal is from alkaline element doping. When the doping concentration is 6 at%, the Curie temperature increases to 55.8 K and the antiferromagnetic phase is undetectable. Continual increase of the doping concentration leads to a decrease of critical temperature. 12 at% K-doped MnO_2_ has the lowest critical temperature (43.7 K). From the M-H measurements, a very weak ferromagnetic-like signal is detected (Fig. 3), thus having the lowest ordering temperature. There are two sharp peaks present in the ZFC curves. This suggests that three transitions may occur in the samples. This phenomenon has been reported elsewhere^18^. The irreversible peak at 24 K is related to spin glass behavior^18^. The peak at 34 K may be related to some disordered structures due to the spin frustration induced by the large amount of K doping, since there is no peak in the FC curve with an applied field as small as 500 Oe^18^. The reverse susceptibility in the inset shows that there is a large amount of antiferromagnetic phase in 12 at% K-MnO_2_. Hence, the ferromagnetic-like ordering is very weak. However, the 16 at% K-doped MnO_2_ has similar ZFC and FC curves to that of 12 at% K-MnO_2_, as shown in the Fig. S6.

### First principles calculations

**Figure 5:**
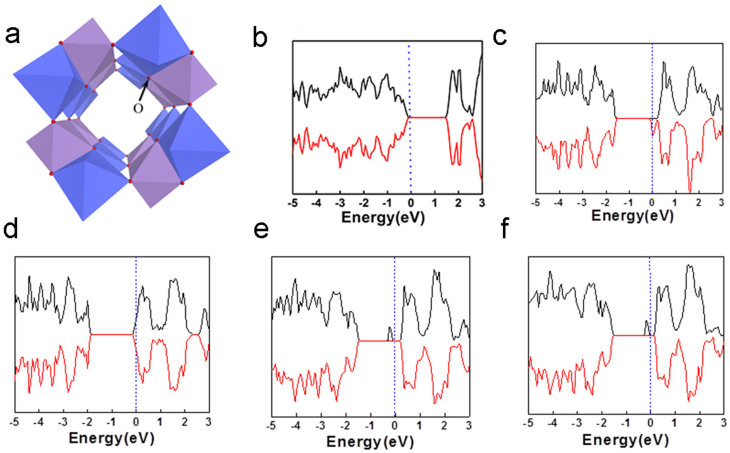
Crystal structure and DOS of alkaline element doped MnO_2_. (a) Staking of MnO_6_ octahedrons in the structures of α-MnO_2_, the oxygen position is shown; (b) DOS of α-MnO_2_ without doping; (c) DOS of 6.25 at% K-doped MnO_2_; (d) DOS of 12.5 at% K-doped MnO_2_; (e) and (f) are the DOS of 6.25 at% Li doped MnO_2_ and 6.25 at% Na-doped MnO_2_.

In order to understand the mechanism of ferromagnetic-like behavior in MnO_2_ nanotubes, we employ first principle calculations to investigate the origin of ferromagnetic-like behavior. For pure MnO_2_, the most stable spin configuration is the antiferromagnetic state as shown by the density of states (DOS) (Fig. 5b), consistent with previous calculations^23^. It shows semiconductor behavior and the Fermi level is inside the energy gap. The bandgap is approximately 1.44 eV, in good agreement with other theoretical and experiments results^23^,^25^. However, after the incorporation of 6.25% K, the spin degeneracy around the Fermi level is broken as shown in Fig. 5c and the entire system shows magnetic properties. The Fermi level is increased to the conduction band, indicating half metallic property.

In this case, one unit cell has a magnetic moment of 1 µ_B_, corresponding to 0.0625 µ_B_/Mn. It is noted that K and O do not show any magnetic moment from our calculations. A saturation magnetization of 5.2 emu/g (at 30 kOe) was experimentally observed in K doped MnO_2_, corresponding to 0.085 µ_B_/Mn, agrees well with theoretical calculations. Further increasing the doping concentration, K ions will be incorporated in neighboring tunnels. The K doping then affects the two different Mn-O octahedrals (purple and blue in Fig. 5a) equally. The magnetic MnO_2_ becomes antiferromagnetic again (Fig. 5d). The difference of the DOS between MnO_2_ without K doping and with 12 at% K doping is the position of Fermi level. The latter one is inside the conduction band, indicating conductive antiferromagnetism instead of semiconductive/insulator antiferromagnetism for MnO_2_ without K doping. Theoretically, if the doping concentration is increased to more than 12.5%, the balance of the homogeneously distributed K atoms may be broken, resulting in the ferromagnetic-like phase again. The stronger ferromagnetic-like ordering in 16 at% K-doped MnO_2_ (Fig. 3b) than that in 12 at% K-doped MnO_2_ was verified by the results of calculations. From our calculations, 18.5 at% K-MnO_2_ shows a half-metallic behavior (Fig. S7 in the supporting information), which means that the antiferromagnetic coupling in 12 at% K-doped MnO_2_ changes to ferromagnetic-like coupling with increasing doping concentration. It is noted that in this case one tunnel of MnO_2_ may contain more than one K ions. The magnetic measurement from these experiments shows that the M-H curve of 12 at% K-MnO_2_ is near linear, indicating that antiferromagnetic coupling dominates. However, a very small coercivity has been observed if the X axis scale is enlarged (Fig. 3b). The minor discrepancy between the experimental and theoretical results may be due to the inhomogeneous distribution of K ions in these samples. Different from the ideal condition of theoretical calculation, in the 12 at% K doping sample, the slightly non-uniform K doping may induce some degree of ferromagnetic-like ordering. However, the main signal from the magnetic measurement by SQUID is antiferromagnetic. From the experimental results analyzed by XAFS, if the doping concentration is higher than 16 at%, the disordered structure was detected, suggesting that K may enter interstitial sites in MnO_2_. Such a disordered structure may result in a paramagnetic behavior. This is different from the periodical model of first principle calculations. Therefore, the sample with higher K doping concentration will not be further discussed in this work.

Similarly, first principle calculations were also employed to calculate Li and Na doped MnO_2_. It shows that both 6.25 at% Li-MnO_2_ and 6.25 at% Na-MnO_2_ show half-metallic behavior, which is similar to that for K doping (Fig. 5e and 5f). Experimentally, it shows that 6 at% Li or Na doped MnO_2_ has very strong ferromagnetic-like ordering at low temperature (i.e. 5 K), which is similar to that 6 at% K-doped MnO_2_, as shown in Fig. 3 and Fig. S5. However, 12.5 at% Li and Na doping in these samples result in the antiferromagnetic coupling again, similar to that of the K-doped MnO_2_. The results have shown that the ferromagnetic-like ordering can be achieved in all the three alkaline elements. The ferromagnetic-like ordering can be tailored by tuning the doping concentration.

**Figure 6:**
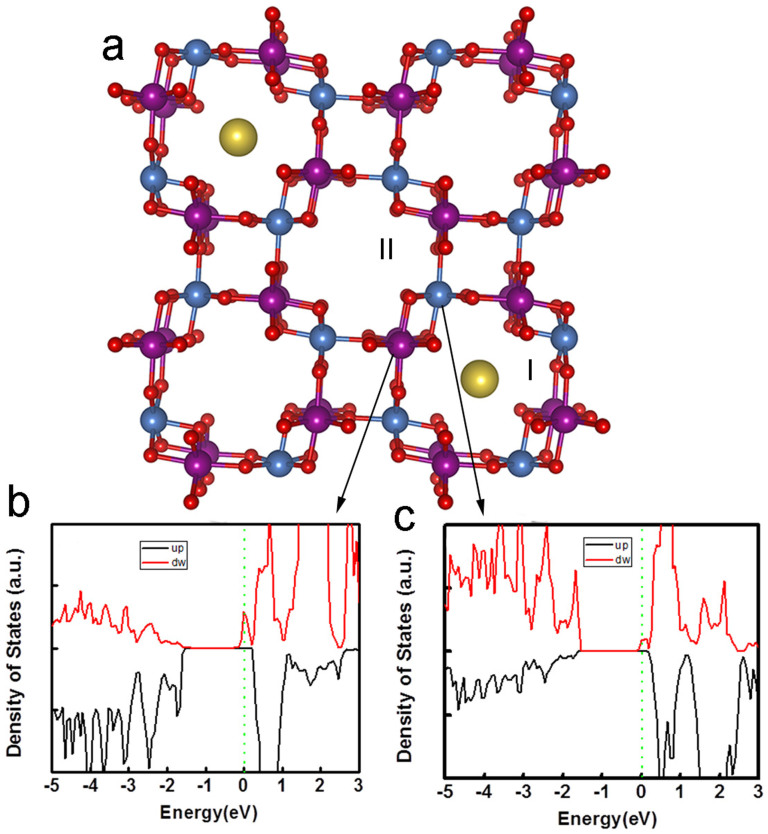
K doping effect on the crystal structure and PDOS of MnO_2_. (a) 2 × 2 tunnel structure of α-MnO_2_ indicating the position of O and Mn. K doping has different effects on two Mn sites (blue and purple) bonded with apex site and plane site of octahedral O respectively; (b) PDOS of Mn bonded with apex site of O; (c) PDOS of Mn bonded with plane site of O.

This doping effect on magnetic properties can be understood by the special crystal structure of α-MnO_2_. α-MnO_2_ is one of the hollandite-romanechite families with 2x2 tunnel structure, similar to β or rutile-MnO_2_. A Mn-O octahedral is expected in every side of tunnel (as shown in Fig. 5a). The two Mn-O octahedrals (blue and purple shown Fig. 5a) are equivalent in the pure α-MnO_2_ and share the O atoms each other. For O atoms in α-MnO_2_, each O atom is shared by two Mn-O octahedrals and occupies the different sites of these two octahedrals: the apex of one octahedral and plane corner of another. Thus, the change of such an O atom has a different effect on these two octahedrals and also the neighboring Mn atoms. If one tunnel is doped with K ions (I in Fig. 6a) while the neighboring tunnel is undoped, i.e. empty (II in Fig. 6a), the interaction between K and such O atom breaks the symmetry between blue and purple Mn-O octahedrals, which ensures the antiferromagnetic coupling between neighboring Mn atoms in pure MnO_2_. Although the geometrical distortion and charge transfer are only minor due to such doping, symmetry breaking of two kinds of Mn-O octahedral transforms the antiferromagnetic state of pure MnO_2_ into ferromagnetic-like state. Fig. 6b and Fig. 6c show the partial DOS (PDOS) projected on to the blue and purple Mn atoms (belong to two different kinds of octahedrals) close to the K atoms after doping. The remarkable difference of the two PDOSs can be observed. The blue Mn atom has a much larger magnetic moment (3.03 µ_B_) than that of the purple Mn (2.9 μ_B_) around the Fermi level. Hence, the symmetry between the purple and blue Mn-O octahedrals is broken, resulting in ferromagnetic-like properties observed in the experiment^31^. Our further calculation (Fig. S7) indicates that the ferromagnetic-like ordering is a combined effect of K doping induced lattice distortion and small charge transfer between K and Mn. The distortion of the lattice leads to the asymmetry of energy splitting and the small charge transfer leads to the overlap of the Fermi level in the conduction band. Because the doping concentration is low, the overall valence state change is very small. For example, for the 6 at% K-doped MnO_2_ sample there will be charge transfer of 6 electrons to MnO_2_ given that the cell size has 100 Mn atoms. Then, the transferred electrons will change the Mn valance from 4+ to +3.94, which is a very small change that may not be detected by XAFS examination.

### Conclusion

We have synthesized K-MnO_2_ with different doping concentrations using a hydrothermal method with KMnO_4_ as the precursor. K-doped MnO_2_ nanotubes with doping concentration lower than 12 at% show ferromagnetic-like ordering. 6 at% alkaline ions doping in MnO_2_ leads to the maximum saturation magnetization. Doping concentration higher than 12 at% leads to disordered structure since some of K ions may enter the interstitial sites due to the higher doping concentration. Li and Na doping also lead to ferromagnetic-like behavior. The results of first principle calculations are consistent with the experimental data. The ferromagnetic-like ordering is due to incomplete filling of K ions in the tunnels, which affects the symmetry of Mn plane, forming ferromagnetic-like ordering.

## Methods

### Synthesis and characterization

α-MnO_2_ nanotubes were prepared using a hydrothermal method, similar to that previously reported^18^,^19^. KMnO_4_ was used as the precursor and was dissolved in HCl solution for 12 hours at 413 K. All chemicals in this work were purchased from Sigma-Aldrich with a purity of 99.99%. The prepared α-MnO_2_ nanotubes contain approximately 8 at% K that was measured using inductively coupled plasma (ICP, ICPMS, PerkinElmer quadrapole Nexion ICPMS) analysis. In order to control the K doping concentration in the MnO_2_ nanotubes, the prepared MnO_2_ nanotubes were soaked into 1 M HCl or 1 M KOH solution for different durations from 1 hr to 6 hrs at 413 K. The nanotubes soaked in the KOH solution showed an increase in the K doping concentration. On the other hand, the nanotubes soaked in the diluted HCl solution resulted in a decrease in doping concentration. It was found that K doping concentration is proportional to soaking time. Through the control of the soaking time combined with the ICP analysis, the K doping concentration was estimated to be approximately 2 at%, 6 at%, 8 at%, 12 at% and 16 at% in atomic ratio respectively. Similarly, 1 M NaOH and 1 M LiOH solutions were also used for the doping process for Li or Na, respectively. MnO_2_ nanotubes were first heated in a diluted HCl solution for more than 24 hrs at 413 K. The amount of residual K in the MnO_2_ tubes was reduced and eventually could not be detected by energy disperse X-ray spectroscopy (EDX) attached to a scanning electron microscope. After soaking the nanotubes in either 1 M NaOH or 1 M LiOH, K ions could not be detected by ICP. The concentration of Li was subsequently measured with ICP. In this work, we prepared Li/Na doped samples with a concentration of approximately 6 at% by controlling the soaking time. X-ray diffraction (XRD, PANalytical Xpert Multipurpose X-ray Diffraction System, Cu Kα radiation), scanning electron microscopy (SEM, FEI Nova NanoSEM 230) and transmission electron microscopy (TEM, JEM-2010, JEOL) were used for the characterization of phases and microstructures. A superconducting quantum interference device (SQUID, Quantum Design XL-5) was used for magnetic property measurements. X-ray absorption fine structure (XAFS) spectra were measured in transmission mode at the XDD beamline at Singapore Synchrotron Light Source (Singapore).

### First principles calculations

First-principles calculations were performed using density functional theory from Vienna ab initio simulation package with a plane wave basis^32^. The generalized gradient approximation (GGA) with spin-polarized Perdew–Burke–Ernzerhof (PBE)^33^,^34^ scheme was employed for calculating the exchange and correlation functional. The core electrons were represented by the projector-augmented-wave (PAW) potential. Kinetic energy cutoff was set at above 400 eV and k-point sampling on the unit cell was 2×2×10. The structure optimization was performed with the criteria of force convergence at 0.01 eV/Å. The optimized lattice constant for α-MnO_2_ is a = b = 0.96 nm and c = 0.28 nm, which is in agreement with previous experimental measurements^35^,^36^,^37^.

## Author Contributions

J.Y. and L.T. did the characterization of materials. H.F. prepared the samples. Y.L. performed the first principle calculations. X. L. did XRD analysis. T.L. did the XAS analysis. M. P. carried out TEM analysis. J. Y. drafted the manuscript. Y. L. drafted the part of calculations. Y.W. revised the part of manuscript on calculations. L.T., S. L. and M.P. gave revision of the manuscript.

## Supplementary Material

Supplementary Information Magnetic properties in α-MnO2 doped with alkaline elements
