# Supplementary material for: Magnetic properties in α-MnO2 doped with alkaline elements
Source: Sci Rep. 2015 Mar 13;5:9094. doi: 10.1038/srep09094 (PMC4358028; doi:10.1038/srep09094)
Supplement: Supplementary Information — Magnetic properties in α-MnO2 doped with alkaline elements [file srep09094-s1.pdf]

Supporting information:

Magnetic properties in α-MnO_2 doped with alkaline elements

Li-Ting Tseng[1], Yunhao Lu[2] , Hai Ming Fan[3] , Yiren Wang[1], Xi Luo[1], Tao Li[4], Paul Munroe[1],

Sean Li[1], and Jiabao Yi[1]*

[1]*School of Materials Science and Engineering, University of New South Wales, Kensington, 2052, NSW, Australia*
[2]*Department of Materials Science and Engineering, Zhejiang University, China*
[3]*Shanxi Key Laboratory of Degradable Biomedical Materials, School of Chemical Engineering, Northwest University, Xi'an, Shanxi 710069, China.*
[4]Institute for Synchrotron Radiation. Karlsruhe Institute of Technology, Karlsruhe, Germany

Email: Jiabao.yi@unsw.edu.au; Tel: 61-293854837; Fax: 61-293856565

Table SI: Coordinate numbers and atomic distance of MnO_2 doped with alkaline elements.

samples	Mn-O			Mn-Mn		
	CN	R	DW	CN	R	DW
Li:Mn	5.8	1.90	0.0043	4.3	2.86	0.0051
Na:Mn	5.9	1.90	0.0045	4.4	2.86	0.0053
K1:Mn	4.6<br>2.2	1.90<br>1.76	0.0008<br>0.001	4.8	2.87	0.0076
K3:Mn	8.6<br>2.9	1.86<br>1.66	0.0038<br>0.0013	5.7	2.89	0.0079
K4:Mn	7.5<br>3.4	1.86<br>1.67	0.001<br>0.001	4.5	2.86	0.0062
K60:Mn	4.5	1.90	0.001	6.7	2.82	0.0114

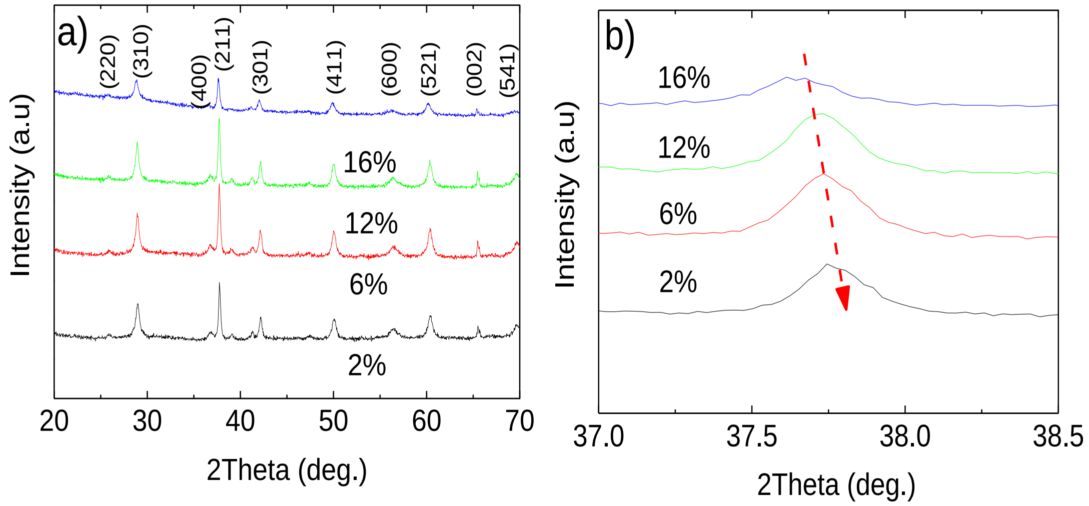

Figure S1: XRD spectra of K doped MnO2 with different doping concentrations. (a) XRD patterns of K-MnO_2 in full range; (b) Zoom in of (a) at the index of (211).

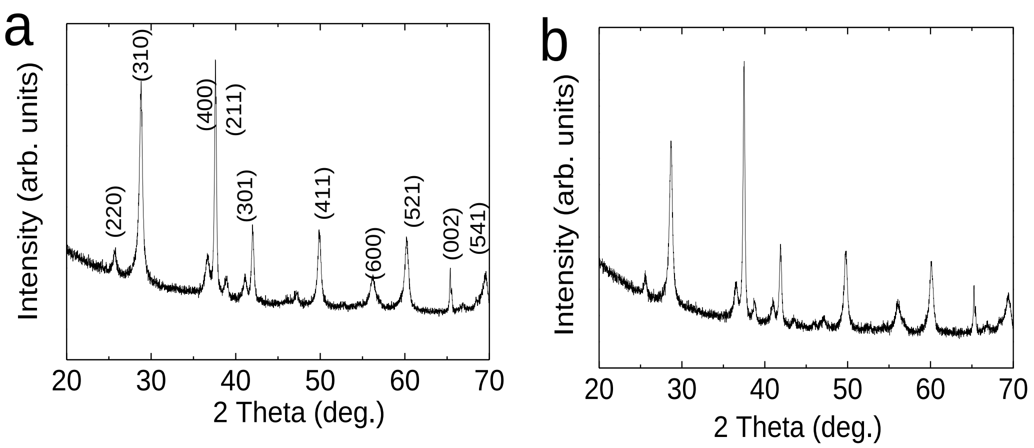

Figure S2: XRD spectrum. (a) 6 % Na doped MnO_2; (b) 6 % Li doped MnO_2.

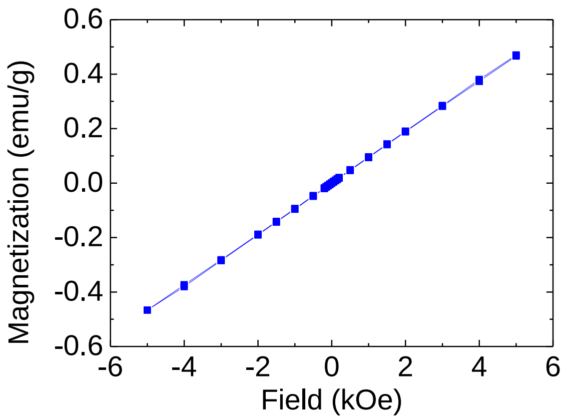

Figure S3: M-H curve of 12% Na doped MnO_2 taken at 5 K.

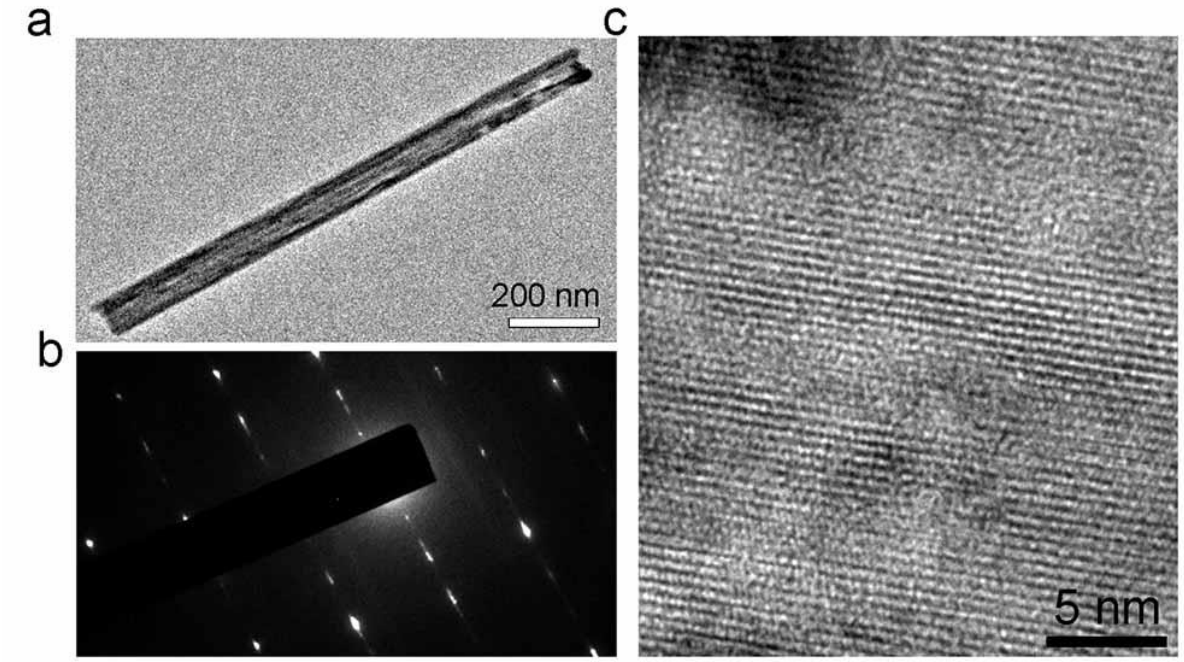

Figure S4: TEM image and SAED of 16% K-MnO_2 nanotubes. (a) TEM image of MnO_2 nanotubes at low magnification; (b) SAED; (c) High resolution TEM image.

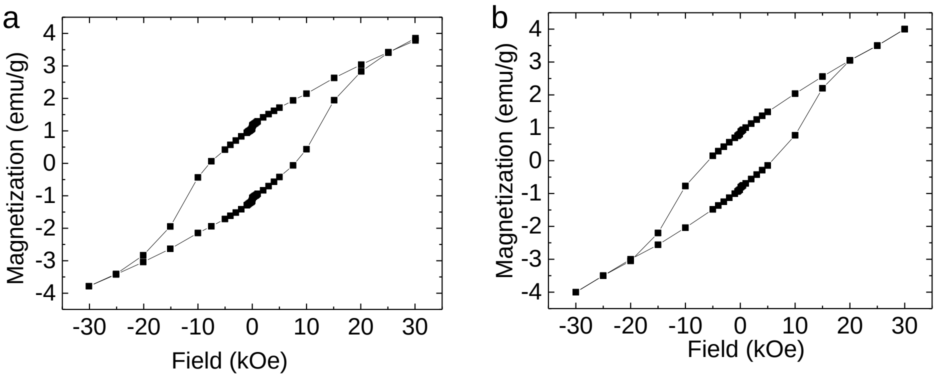

Figure S5: M-H loops taken at 5 K. (a) 6% Li-MnO_2; (b) 6% Na-MnO_2.

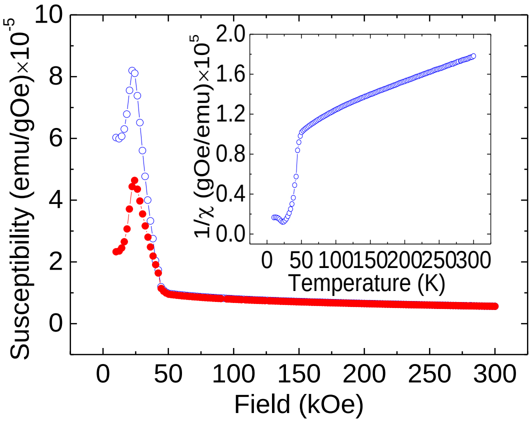

Figure S6: ZFC and FC curves of 16% K doped MnO_2 nanotubes.

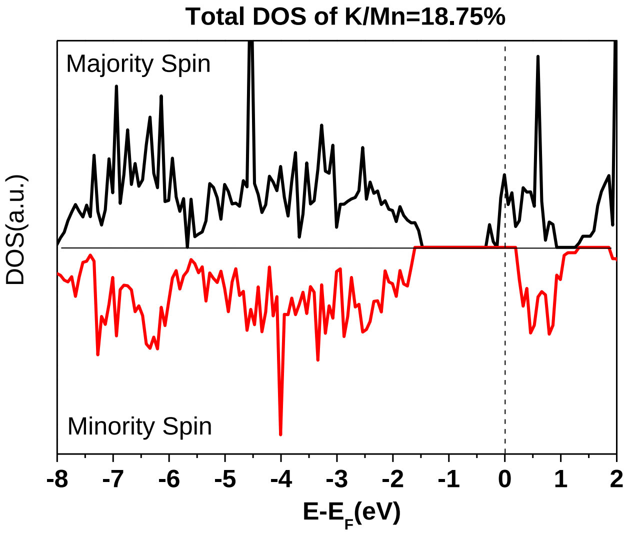

Figure S7: DOS of 18.5% K doped MnO_2, indicating half-metallic behaviour.

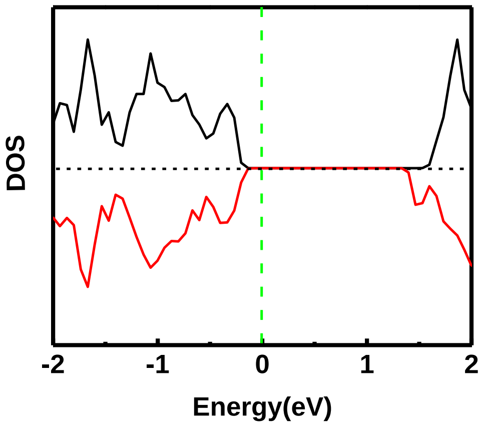

Figure S8: DOS of K doped MnO_2 without charge transfer. From the calculation, it can be seen that the Fermi level is inside the bandgap. Although the spin up and spin down in the conduction band is asymmetric, there are no filled states close to the Fermi level. Hence, the overall system is non-magnetic. This calculation has demonstrated that charge transfer is indispensable for the formation of ferromagnetic ordering.
